# Cortical auditory-evoked potential as a biomarker of central auditory maturation in term and preterm infants during the first 3 months

**DOI:** 10.6061/clinics/2021/e2944

**Published:** 2021-10-05

**Authors:** Dayane Domeneghini Didoné, Lilian Sanches Oliveira, Alessandra Spada Durante, Kátia de Almeida, Michele Vargas Garcia, Rudimar dos Santos Riesgo, Pricila Sleifer

**Affiliations:** I Programa de Pos-Graduacao em Saude da Crianca e do Adolescente, Universidade Federal do Rio Grande do Sul (UFRGS), Porto Alegre, RS, BR.; II Faculdade de Ciencias Medicas, Santa Casa de Sao Paulo, (FCMSCSP), Sao Paulo, SP, BR.; III Universidade Federal de Santa Maria (UFSM), Santa Maria, RS, BR.; IV Departamento de Saude e Comunicacao Humana, Universidade Federal do Rio Grande do Sul (UFRGS), Porto Alegre, RS, BR.

**Keywords:** Auditory-Evoked Potential, Premature Infant, Infant, Child Development, Electrophysiology

## Abstract

**OBJECTIVES:**

To analyze central auditory maturation in term and preterm infants during the first 3 months of life by comparing the latency and amplitude of cortical auditory-evoked potential at different frequencies.

**METHODS:**

In this study, 17 term and 18 preterm infants were examined; all had tested positive on the neonatal hearing screening test. Cortical auditory potential was investigated during the first and third months of life. The response of the cortical auditory-evoked potential was investigated at frequencies of 500, 1000, 2000, and 4000 Hz. The latency and amplitude of the cortical response were automatically detected and manually analyzed by three researchers with experience in electrophysiology. The results were compared using analysis of variance and the Bonferroni test. A significance level of 5% was used for all analyses.

**RESULTS:**

Latency values of cortical auditory-evoked potential in the first month of birth were significantly higher than those in the third month, and latency values of the preterm group were higher than those of the term group, regardless of the frequency and time of evaluation. In general, the latency of the cortical auditory-evoked potential was higher at high frequencies. Amplitude values in the third month of life were significantly higher than those in the first month for term and preterm infants.

**CONCLUSION:**

Central auditory maturation was observed in both groups but with different results between those born at term and preterm, with latencies of cortical auditory-evoked potential higher for the preterm group and at high frequencies.

## INTRODUCTION

According to the World Health Organization (1,2), the estimated preterm birth rate was approximately 9.8% in 2000 and 10.6% in 2014, with preterm birth being the leading cause of death in children younger than 5 years. Of those who survive, many are afflicted with learning, visual, and auditory problems (1,2). Even in the absence of brain lesions, cognitive and language problems are common. One in three children has language abnormalities (3); therefore, they should be monitored to identify early hearing loss and/or central auditory changes (4), including changes related to the maturation process of structures related to hearing (5).

Central auditory maturation may be impaired owing to preterm birth. The third trimester of gestation is important for the maturation of the auditory system. With preterm birth, this process is interrupted and continues in the extrauterine environment, but whether this accelerates or delays the development of the auditory pathways remains unknown (6).

Therefore, the electrophysiology of hearing is of great importance in the stages of child development because it allows clinicians to understand and monitor the maturation of auditory structures (7). Although studies of brainstem auditory-evoked potential are of great value for understanding these conditions and providing evidence of differences in the maturational pattern of preterm births (6), the use of long-latency auditory-evoked potential allows the verification of conditions of the central auditory system, making this evaluation an additional tool in the study of auditory maturation. Some studies have been conducted on preterm births (3,8-10) to assess conditions of the central nervous system in this population (9).

Cortical auditory-evoked potential (CAEP), represented by the complex P1-N1-P2 potential, is part of the long-latency auditory-evoked potential. In small children, the P1 component is mainly visualized in tracing and has been considered a neurophysiological biomarker of auditory development (10,11). This auditory potential can be evoked with different acoustic stimuli, from pure tones to speech stimuli, depending on the researcher’s objectives. Pure tone stimuli may reflect the organization and cortical development of different central auditory areas essential in the investigation of maturation (12), as the auditory cortex is organized in a tonotopic manner (13).

Early detection of functional alterations in the auditory system in populations considered at risk, such as preterm infants, is crucial for devising intervention strategies (5). However, the use of CAEP for examining auditory maturation in infants is still recent and requires additional research, especially for populations considered at risk of alterations in the development of auditory pathways. Studying CAEP allows access to the functionality of central auditory structures, and it can be used to investigate the maturation of auditory pathways.

Currently, research involving the study of CAEP in the infant population and the use of automatic analysis devices have helped obtain results, especially for young children, as responses are influenced by brain maturation and are often challenging to visualize (14). Therefore, in very young children, the automatic analysis of cortical responses may help clinicians interpret the results because of the great influence of the maturation process on the morphology of CAEP.

The study of the maturation of the auditory pathway in preterm infants is necessary and provides information to clinicians about the development of auditory pathways in this population. The P1 cortical potential is considered a biomarker of central auditory development and is a promising marker for understanding the neurophysiological basis of hearing. Therefore, understanding the functioning of central auditory pathways of preterm infants would help clinicians make informed decisions about interventions for this population.

Based on the above mentioned statements, the objective of the present study was to analyze central auditory maturation through cortical potential in term and preterm newborns during the first three months of life by comparing the latency and amplitude at different frequencies.

## MATERIALS AND METHODS

This is a longitudinal and observational study conducted by the Universidade Federal do Rio Grande do Sul in partnership with the Faculdade de Ciências Médicas da Santa Casa de São Paulo (FCMSCSP). This study was approved by the Research Ethics Committees of both universities (44965015.8.1001.5334 and 51349315.6.1001.5479, respectively). This project was conducted in accordance with the ethical procedures recommended by Resolution 466/2012 of the National Health Council. Only newborns and infants were included in this study. Parents or guardians agreed to the procedures and signed an opt-out consent form.

The study participants were infants born at term and preterm. Participants were selected for convenience from the outpatient clinic and neonatal intensive care unit (NICU). The medical records of all participants were evaluated to verify the inclusion and exclusion criteria. Participants were assessed during the first and third months of life to verify aspects of central auditory maturation during this developmental period.

The following inclusion criteria were adopted for the term birth group: gestational age ≥37 weeks, absence of risk indicators for hearing loss according to the Joint Committee on Infant Hearing (2019) (4), good health, a positive result on the neonatal hearing screening test, electrophysiological record of adequate morphological quality, and cooperation during the test. The following inclusion criteria were adopted for the preterm birth group: gestational age ≤36 weeks, good health, a positive result on the neonatal hearing screening test, electrophysiological record of adequate morphological quality, and cooperation during the test. Subjects with hearing impairment syndromes, family history of hearing impairment, congenital anomalies, neurological disorders, congenital infection, bacterial meningitis, and blood transfusion were excluded from the preterm group.

Initially, 114 participants’ families were contacted, and 48 did not agree to participate. During the first month, 66 neonates were examined. Of them, 37 were examined again during the third month of life. Owing to the inability to undergo the examination, two infants were excluded, and the final sample consisted of 17 term infants (control group) and 18 preterm infants (study group). The participants comprised both sexes and had bilateral positive results on the neonatal hearing screening test, determined using transient-evoked otoacoustic emissions (TEOAEs) (15) and/or automated brainstem auditory-evoked response procedures performed prior to hospital discharge (4).

In preterm birth group, one participant was considered extremely premature, 10 moderately premature, and 7 borderline premature. A previous statistical analysis (analysis of variance, ANOVA) did not show differences between different gestational age classifications for the variables analyzed in this study; therefore, preterm birth infants constituted a single group.

For the first stage of the evaluation, participants in both groups were examined between 38 and 43 weeks of gestation, and for the second stage, between 49 and 57 weeks, considering the mother’s last menstrual period. Age correction was performed considering 38 weeks as a reference to consider the term ‘newborn’ (1,2), as this was the minimum gestational age for term infants. Thus, based on other studies (9,16), the objective of this correction was that neonates of both groups were of the same postconceptional age.

The group of term births was considered as the control, as responses were in accordance with those described in another study with the same equipment involving this population (17).

All subjects underwent CAEP assessments during the first and third months of life. The cortical potential P1 was analyzed and characterized by a peak between 150 and 400 ms. Only a positive peak was observed on electrophysiological tracing, reflecting characteristics of the acoustic stimulus (tone burst).

The Hearlab System (National Acoustic Laboratories) was used to analyze CAEP. The evaluation was performed for each participant on only one side, and the same side was maintained between the first and second evaluations because the equipment had one channel and did not allow simultaneous evaluation of both ears. The side chosen was based on the position of the neonate in the lap of the mother, considering the comfort of both. A previous statistical analysis of the findings of participants assessed on the right side and those assessed on the left side did not show significant differences. Thus, participants of each group (preterm or term) were included within the same group, regardless of the evaluated side. Similar protocol and results have also been described in another study (17).

The choice of stimulus type was owing to the availability of equipment and because the use of tone burst stimuli can reflect the organization and cortical development of different central auditory areas essential in the maturational investigation (12).

The cortical auditory potential was assessed in an acoustically and electrically treated room. The parents or guardians sat in a comfortable chair, with participants comfortably positioned on their laps. The ambient noise did not exceed 35 dB (A). The equipment had been previously calibrated by a qualified professional according to technical specifications.

To perform the electrophysiological study, neonates were maintained in a light sleep during the first month of life (17), whereas infants were kept alert during the third month of life. Brazelton scale (1973) (18) was used to guarantee the behavioral status of newborns. The alert state during the third month was maintained through play instruments presented to the children. In the first month of life, a previous analysis was performed with 15 neonates in light sleep and 10 on alert, and no significant difference was observed for the variables analyzed (*p* *-*value on ANOVA >0.4). Because of this and that neonates spend most of their time in light sleep, they were examined in this behavioral state during the first month of life. The parameters used to assess cortical potential P1 are described in Table 1 (19).

In this study, only a trace was defined for each stimulus because the equipment used automatic analysis and did not allow the visualization of two tracings simultaneously. This protocol was in accordance with previous recommendations (20).

The presence or absence of responses at each frequency was automatically detected by the equipment. Regarding detection, the equipment considered a positive component with a latency interval between 0 and 500 ms as a response. After detecting this component, the equipment performed a statistical analysis of the responses. For statistical analysis, the equipment applied the Hotelling’s T2 statistical test. Each P1 component formed was analyzed by comparing the noise level and the response obtained; that is, if the response was consistent and different from the noise, it was considered significant.

Thus, the equipment informed the examiner the response and whether it was significant, that is, if the response was maintained throughout the evaluation and whether it differed from the noise. The latency and amplitude were marked by three examiners.

**Figure 1:**
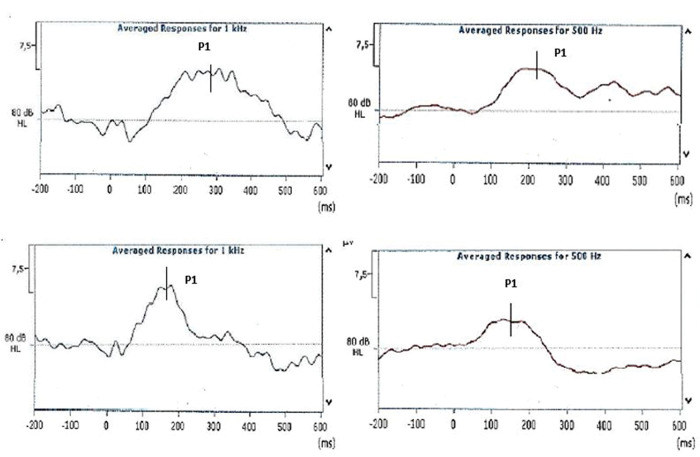
Example of cortical potential P1 in the first (superior images) and third months of life (inferior images).

The examiners were instructed to mark P1 at the highest positive peak observed within the 500-ms window. The evaluations were performed with examiners blind to patient group data. There was full agreement between markings of the examiners. After the analysis, the latency and amplitude of component P1 were marked on the equipment by the main researcher. The latency was considered at the point of greatest amplitude of the P1 component, based on examiners’ markings. The amplitude was defined from the baseline, that is, the measure between the baseline and peak of the maximum wave. Figure 1 shows the responses obtained during the first and third months of life.

Data were tabulated in Excel spreadsheets and analyzed using the Statistical Package for Social Sciences program, version 20.0. The following statistical tests were used: Student’s *t*-test, chi-square test, ANOVA, and Bonferroni test. A significance level of 5% was adopted for all analyses.

## RESULTS

Data described in this sample are listed in Table 2. There was no significant difference in age in the assessment period between groups and the distribution of participants in relation to sex and assessed ear.

**Figure 2:**
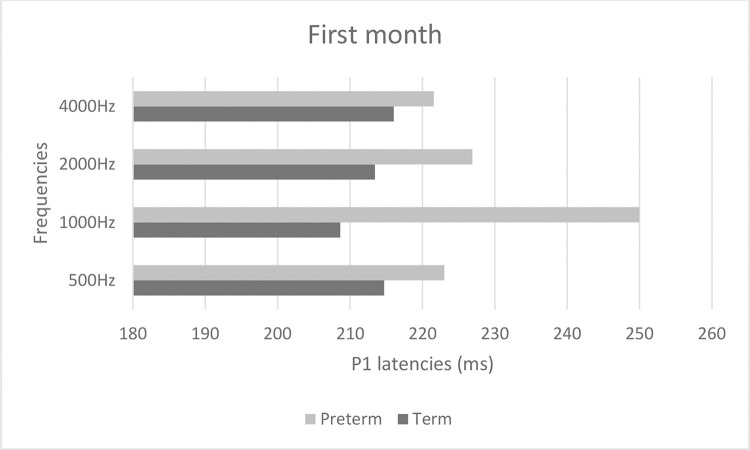
P1 latency values in term and preterm infants in the first month of life.

**Figure 3:**
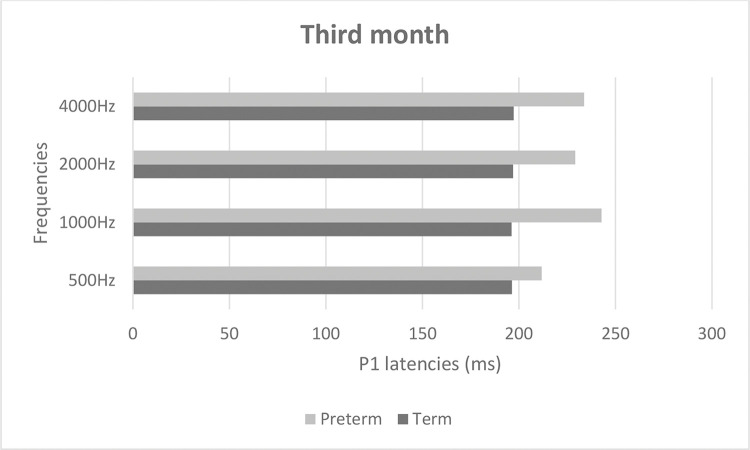
P1 latency values in term and preterm infants in the third month of life.

Figures 2 and 3 show mean latencies of the P1 component in term and preterm infants during the first and third months of life, respectively. In both the first and third months, the latency of the P1 component was higher in preterm than in term infants.

Table 3 shows that latency values in the first month were significantly higher than those in the third month (*p*<0.001), regardless of the frequency and group. In addition, latency values of the preterm birth group were higher than those of the term group, regardless of the frequency and time of evaluation (*p*<0.001). When comparing latency values of the P1 component, the latency was higher at high frequencies in both groups (Table 3).

Table 4 shows that amplitude values of the P1 component in the third month were significantly higher than those in the first month (*p*<0.001), regardless of the frequency and group.

## DISCUSSION

In this study, it was possible to assess central auditory maturation during the first 3 months of term and preterm infants through CAEP P1 for different tonal stimuli. This potential is a neurophysiological biomarker that is feasible in the study of typical and atypical auditory development in children (7,10,21).

A decrease in latency values was observed for all frequencies used to evoke the P1 component from the first to the third month of life in both groups. These results corroborate the scientific literature, which reports that this is the main component visualized in the tracing of children and decreases its latency according to central auditory maturation (10,21). It is known that auditory cortical potential is generated in the temporal auditory cortex (22). Thus, latency modifications refer to the faster processing of the sound stimulus in the primary auditory cortex as a function of myelination of the auditory pathways.

Despite the decrease in latency of the P1 component, the maturation of central auditory structures was different between groups because it was verified that the latency values of the preterm group were higher than those of the term group. This suggests typical development in the term group and slower maturation in the preterm group in the present study, despite the corrected age. These results corroborate those of a study that showed evidence of changes during the maturation process of preterm births through CAEP (3). These results also support those of another study (23), in which the authors analyzed different cortical auditory potential in the first and third months of life between term and preterm infants. The authors also described greater differences in latencies of potential during the third month of life, suggesting central auditory system immaturity in the preterm group, despite the corrected age.

In a literature review, authors (5) mentioned a study that pointed out changes along the developmental timeline of preterm infants. The results of this study also agreed with a recent meta-analysis (6), in which the authors analyzed studies with brainstem auditory-evoked potential of term and preterm newborns evaluated at 37-46 weeks of gestational age (term). The authors verified that differences exist in the maturation process of these groups, and preterm neonates present delays in the intervals of brainstem auditory-evoked potential, reflecting atypical maturation along the auditory pathway. Researchers also believe that this immaturity possibly extends to central auditory levels, a fact that was verified in the present study.

In contrast, a recent study (10) showed lower latency values of P1 in preterm infants during the third month of life, showing rapid cortical auditory development in this population. These differences can be explained by differences in the categorization of samples between studies. In addition, it is known that the environmental acoustic exposure of preterm infants may have been different between studies, favoring better cortical development.

In relation to the frequency comparison, the latency of the P1 component was higher at high frequencies in both groups. The latency values of the 4000-Hz frequency were significantly higher than those of the 500-Hz frequency in the preterm group. Reportedly, the ability of the central auditory nervous system to discriminate pure tones at 4000 Hz is worse than that at 500 Hz, before the age of 3 months. After this age, this is reversed, and children tend to discriminate pure tones of 4000 Hz better than those of 500 Hz (24). In addition, hearing discrimination ability tends to improve with increasing age (25). Thus, the higher latency with 4000 Hz when compared with 500 Hz may be related to the age range of the participants, which was until 3 months of life in this study (24).

For the term group, the 1000-Hz latency in the first month of life was significantly higher than the 500-Hz latency in the third month of life, suggesting maturation of central regions related to 500 Hz. Other studies (12) also showed better responses of cortical potential when evoked from even lower frequency stimuli than from high frequencies, justifying the findings of this study. In addition, the latency value at 4000 Hz was higher than that at 2000 Hz in the first month of life and all frequencies in the third month. This result complements others, because in this study it was evident that the cortical auditory responses to higher frequencies had a higher latency than those to lower frequencies, especially in the first month of life, when the central nervous system’s auditory immaturity is more evident, in agreement with previous studies (12,24).

When frequency analysis was performed between groups, latency values of preterm neonates at frequencies of 1000 and 4000 Hz were higher than those of term neonates compared to the frequencies of 500 Hz; that is, there is also a maturational difference between groups when the comparison is performed based on frequencies, with the latency of the P1 component being lower at lower frequencies in preterm neonates. As already explained, the preterm infants in this study presented with a delay in maturation when compared with term infants, which justifies these results. However, no studies have compared these differences based on frequency. It is believed that this difference between groups may be related to the possible central tonotopic disorganization of this population at this age. Preterm infants are usually exposed to environments such as the NICU, which often present a reduced quality of acoustic stimulation, compromising neuroplasticity of the auditory system (5). In this study, some newborns remained in NICU—this may have influenced the results. This is because overexposure to high frequency noise in this period of development can disrupt the functional organization of auditory cortical circuits and influence central auditory maturation (26); this, together with the maturation issue, may explain the worse results at high frequencies.

In relation to the amplitude of the P1 component, a similar behavior was observed in both groups during the first and third months of life. An increase was observed from the first to the third month at all frequencies evaluated. This fact corroborates that of a study (21) that also described increased amplitude for the P1 potential in the first months of life. In this study, amplitude values did not differ between groups. This result is believed to be owing to the large variation in P1 amplitude in groups. Thus, in this study, latency values were more reliable for predicting maturational differences between term and preterm infants.

From the present research, it was possible to verify that the maturation of central auditory structures can be studied through the cortical potential P1, which is an additional tool. In addition, differences were observed in maturation processes of term and preterm births during the first 3 months of life, suggesting, from all described results, that the maturational velocity at the central level could be slower in preterm births, even at the corrected age (8).

Further studies during the first years of life are needed to better understand the impact of these observed differences up to 3 months of life. Longitudinal studies may verify the spontaneous recovery of these responses during development or whether they are already predictive of changes in central auditory processing.

## CONCLUSION

Central auditory maturation was observed in both groups but with different results between those born at term and preterm, with latencies of P1 higher in the preterm group and at high frequencies.

## AUTHOR CONTRIBUTIONS

Didoné DD, Durante AS, Riesgo RS and Sleifer P were responsible for the study conception. The original manuscript draft was written by Didoné DD. Didoné DD and Oliveira LS were responsible for the investigation and methodology. Durante AS, Garcia MV, Riesgo RS and Sleifer P edited and reviewed the manuscript. Durante AS and Almeida K were responsible for the project administration. Durante AS, Almeida K, Garcia MV, Riesgo RS and Sleifer P supervised the study.

## Figures and Tables

**Table 1:** Parameters used to assess the auditory cortical potential P1.

Module	Cortical Tone Evaluation (CTE)
Evaluated frequencies	500, 1000, 2000, and 4000 Hz
Intensity	80 dBHL
Polarity	Alternating
Interval between stimuli	1.125 ms
Stimulus rejection	<20%
Residual noise	≤3.6 μV
Transducer	Insertion headphones ER-3A
Stimulus speed	0.5 Hz
Duration	40 ms
Rise-fall	10 ms
Plateau	30 ms
Filter	High-pass: 0.16 Hz; Low-pass: 0.30 Hz.
Envelope	Cosine
Signal amplification	1.210
Positioning of electrodes	Active electrode: Cz; Ground electrode: Fpz; Reference electrodes: M2 or M1
Pre-stimulation	-100 ms
Window	600 ms
Number of stimuli	Minimum 50 and maximum 200
Response analysis	Automatic/Objective (present when *p*≤0.05)
Marking responses (latency and amplitude)	Manually considered by three examiners with experience in electrophysiology
Impedance	<5 KΩ

dBHL, decibels Hearing Level.

**Table 2:** Characterization of the sample.

Variables	Total sample (n=35)	Term (n=17)	Preterm (n=18)	*p*-value
Gestational age (weeks)	Average	39.76	33.46	<0.001*
	Minimum	38	29	
	Maximum	41	36	
	Standard deviation	0.90	2.22	
Age at first evaluation (weeks)	Average	41.40	40.08	0.686*
	Minimum	39	38	
	Maximum	43	43	
	Standard deviation	1.24	1.22	
Age at second evaluation (weeks)	Average	52.52	51.94	0.306*
	Minimum	51	49	
	Maximum	55	57	
	Standard deviation	1.37	1.89	
Ear	Right	10	11	0.890**
	Left	7	7	
Sex	Female	8	6	0.407**
	Male	9	12	

*Student’s *t*-test, **chi-square test, *p*≤0.05 is considered significant.

**Table 3:** Comparison of latency values (milliseconds) of P1, according to frequency, period of evaluation, and group.

Factor	Level of Factor	Frequency								*p*-value	Bonferroni test
		500 Hz		1000 Hz		2000 Hz		4000 Hz			
		Average	SD	Average	SD	Average	SD	Average	SD		
Period of evaluation	1^st^	235.90	37.00	237.52	43.29	227.28	29.11	263.66	61.94	<0.001*	1^st^ > 3^rd^
	3^rd^	204.29	37.65	220.14	40.87	213.51	42.40	216.03	46.74	<0.001*	Preterm > Term
Group	Term	214.71	40.70	208.65	30.37	213.44	33.60	221.56	51.91	0.276	
	Preterm	223.03	40.09	249.97	44.10	226.90	40.56	255.80	61.60	0.008*	4000 Hz > 500 Hz
Period of evaluation * Group	1^st^ * Term	233.06	38.66	221.12	25.93	229.94	29.44	245.88	56.08	0.047*	1st 1000 Hz > 3rd 500 Hz; 1st 4000 Hz > 1st 2000 Hz 3rd 500 Hz. 3rd 1000 Hz. 3rd 2000 Hz. 3rd 4000 Hz
	1^st^ * Preterm	239.92	35.78	260.75	52.80	223.50	29.51	288.83	63.33	0.038*	Preterm 1000 Hz > Term 500 Hz. Term 1000 Hz. Term 2000 Hz; Preterm 4000 Hz > Term 500 Hz. Term 1000 Hz. Term 2000 Hz. Preterm 500 Hz
Period of evaluation * Group	3^rd^ * Term	196.35	34.71	196.18	29.99	196.94	29.71	197.24	33.99	0.447	
	3^rd^ * Preterm	211.78	39.72	242.78	37.11	229.17	47.22	233.78	50.94		

*Repeated Measures ANOVA and Bonferroni test; SD: standard deviation; Significance level adopted: *p*≤5%.

**Table 4:** Comparison of amplitude values (microvolts) of P1, according to frequency, period of evaluation, and group.

Factor	Level of Factor	Frequency								Effect	*p*-value	Bonferroni
		500 Hz		1000 Hz		2000 Hz		4000 Hz				
		Average	SD	Average	SD	Average	SD	Average	SD			
Period of evaluation	1^st^	6.97	3.18	7.91	3.71	6.81	2.92	5.78	2.92	Moment of evaluation	<0.001*	3^rd^ > 1^st^
	3^rd^	8.89	3.95	9.21	4.30	9.44	4.99	10.24	5.56	Group	0.186	
Group	Term	7.60	3.72	8.11	4.10	7.89	4.30	7.21	3.79	Moment of evaluation*Group	0.786	
	Preterm	8.49	3.73	9.20	4.02	8.65	4.46	9.37	6.04	Frequency	0.762	
Period of evaluation * Group	1^st^ * Term	6.27	2.99	7.60	3.95	6.78	2.76	5.56	2.45	Frequency*Moment of evaluation	0.067	
Period of evaluation * Group	1^st^ * Preterm	7.95	3.30	8.36	3.45	6.84	3.25	6.10	3.58	Frequency*Group	0.824	
Period of evaluation * Group	3rd * Term	8.93	3.98	8.63	4.29	9.01	5.27	8.86	4.22	Frequency * Moment of evaluation * Group	0.472	
Period of evaluation * Group	3rd * Preterm	8.85	4.04	9.77	4.36	9.85	4.82	11.55	6.44			

*Repeated measures ANOVA and Bonferroni test; SD,: standard deviation; *p*≤0.05 is considered significant.
